# Dynamic interactions of dimeric hub proteins underlie their diverse functions and structures: A comparative analysis of 14-3-3 and LC8

**DOI:** 10.1016/j.jbc.2025.108416

**Published:** 2025-03-17

**Authors:** Jesse Howe, Elisar J. Barbar

**Affiliations:** Oregon State University, Department of Biochemistry and Biophysics, Corvallis, Oregon, USA

**Keywords:** bivalency, dimerization, heterogeneity, hub proteins, intrinsically disordered proteins, multivalency, protein dynamics, protein-protein interactions

## Abstract

Hub proteins interact with a host of client proteins and regulate multiple cellular functions. Dynamic hubs have a single binding interface for one client at a time resulting in competition among clients with the highest affinity. Dynamic dimeric hubs with two identical sites bind either two different client proteins or two chains of the same client to form homogenous complexes and could also form heterogeneous mixtures of interconverting complexes. Here, we review the interactions of the dimeric hubs 14-3-3 and LC8. 14-3-3 is a phosphoserine/threonine binding protein involved in structuring client proteins and regulating their phosphorylation. LC8 is involved in promoting the dimerization of client peptides and the rigidification of their disordered regions. Both 14-3-3 and LC8 are essential genes, with 14-3-3 playing a crucial role in apoptosis and cell cycle regulation, while LC8 is critical for the assembly of proteins involved in transport, DNA repair, and transcription. Interestingly, both protein dimers can dissociate by phosphorylation, which results in their interactome-wide changes. Their interactions are also regulated by the phosphorylation of their clients. Both form heterogeneous complexes with various functions including phase separation, signaling, and viral hijacking where they restrict the conformational heterogeneity of their dimeric clients that bind nucleic acids. This comparative analysis highlights the importance of dynamic protein-protein interactions in the diversity of functions of 14-3-3 and LC8 and how small differences in structures of interfaces explain why 14-3-3 is primarily involved in the regulation of phosphorylation states while LC8 is primarily involved in the regulation of assembly of large dynamic complexes.

A protein’s interactome is defined as the set of interacting partners that the protein can bind (1). Most proteins have small interactomes, but a small group of proteins called hubs interact with a large number of binding clients, making them central in a large web of protein-protein interactions (2, 3). Hubs often regulate diverse cellular processes such that knockouts of hub proteins interfere with function in multiple pathways within the cell, making hub proteins essential regulators of cellular outcomes (4, 5, 6).

Hub proteins can be categorized into two groups based on the surface they use to bind clients (Fig. 1*A*). Static hubs bind multiple clients simultaneously using multiple interfaces, while dynamic hubs have a single binding interface which necessitates competition for binding by clients (7). While the dimeric hubs LC8 and 14-3-3 bind clients at two sites, they are dynamic hubs due to the use of the same binding interface to interact with all clients. Both proteins fit the definition of dynamic hubs because they almost exclusively either bind a single peptide chain at two sites or two copies of identical peptide chains, rather than two different clients, resulting in competition for the binding interface (8).

**Figure 1:**
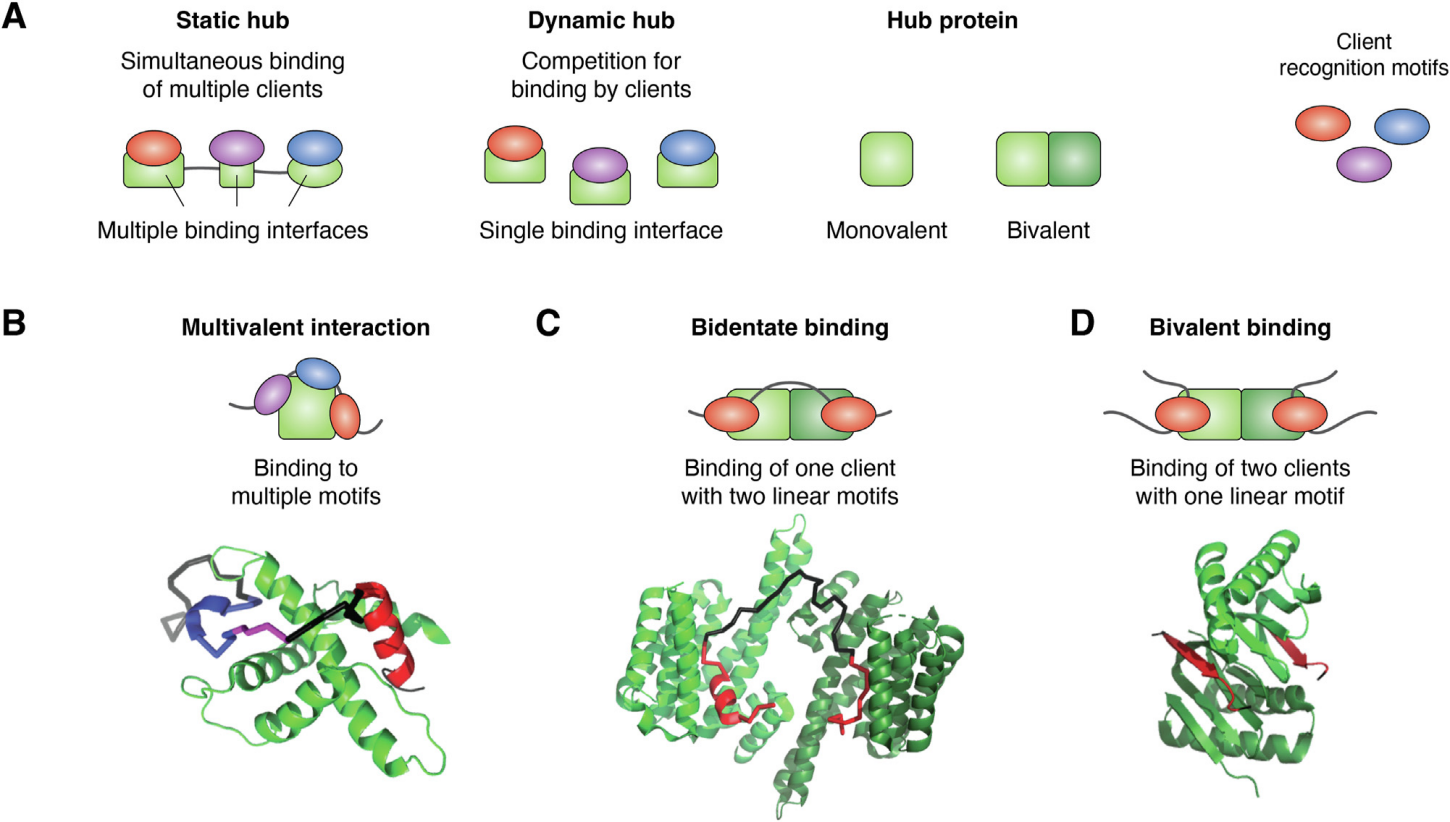
**Structures of hubs and binding modes.***A*, hub proteins bind a variety of partner peptides. Static hubs bind clients at multiple sites along their structure, often simultaneously. Dynamic hubs bind clients at a single binding interface for many clients through spatiotemporal regulation of partner selection. Hub proteins can be monovalent (*dark green*) or bivalent (*dark* and *light green*). *B*, the TAZ domain of CREB binding protein interaction with its client CITED2 is multivalent (PDB code: 1R8U). *C*, 14-3-3 binds clients with high affinity in a bidentate binding mode, as in its interaction with yeast-neutral trehalase (PDB code: 5N6N). Bidentate binding occurs when two sites in a single client are bound by a single dimeric hub. The complex is stabilized by the high local concentration of binding sites linked together in the client. *D*, bivalent binding in which two binding sites in a hub protein each binds a separate chain of the same client protein. LC8 binds clients exclusively in a bivalent mode. Shown is LC8 in complex with two chains of a peptide (*red*) derived from CHICA (5E0L).

One static hub is the antitumor protein BRCA2, which contains multiple DNA and protein binding domains and serves as a scaffold that localizes DNA repair effectors to sites of DNA damage (9, 10). This function is central to its role in DNA repair and homologous recombination. BRCA2 is regulated heavily by posttranslational modification and interaction with numerous client proteins (11, 12). Simultaneous binding to multiple clients allows static hubs to bridge between clients with separate functions. Since BRCA2 binds multiple clients at different interfaces, it differs from dimeric dynamic hubs which most often bind either one peptide or two identical peptides.

Another static hub is the transcriptional coactivator CREB-binding protein (CBP) which binds multiple transcription factors, acting as a scaffold to regulate transcription of a wide variety of genes (13). While CBP contains many protein binding domains, several of these domains such as the TAZ domains also bind many clients at the same binding interface (14, 15) (Fig. 1*B*). For example, the TAZ domain of CREB binds the transcription factor hypoxia-induced factor 1a (HIF-1a), which is displaced from TAZ upon introduction of CITED2, the negative regulator of HIF-1a (16, 17). Both HIF-1a and CITED2 are disordered and form ordered structures upon binding to the TAZ domain. These domains can be thought of as dynamic hubs since they bind multiple clients at a single interaction surface.

The binding diversity of dynamic hubs derives from their tendency to bind disordered proteins at short linear motifs (SLiMs) and the structural plasticity of their binding interfaces (2). A classic example of a dynamic hub protein is calmodulin (CaM), which binds >300 clients with a highly flexible binding interface (18). CaM contains two independently folded domains connected by a helical linker region (19). Each folded domain can bind two Ca^2+^ ions, which results in a conformational change that leads to high affinity binding to client proteins (20, 21). The ability of CaM to form diverse structures in complex with its intrinsically disordered binding partners is due to the folding of these clients into a helical conformation and the complementary structural changes in CaM upon binding (18). CaM can also act as an adaptor protein that binds two different clients linking them together (22).

Here we review the cellular functions, structures, and interactions of two well-characterized dimeric hub proteins, 14-3-3 and LC8. 14-3-3 is a dimeric phosphoserine/threonine binding protein with over 300 known clients involved in various cellular pathways (23). LC8 is a dimeric protein that functions in a number of biological processes binding over 100 clients and promoting their dimerization (1). While both 14-3-3 and LC8 interact bivalently and share some conserved functions such as in viral hijacking, their different structures and binding stoichiometries and mechanisms explain the diversity of their functions. We also highlight the importance of the heterogeneity of hub-client complexes in regulating client function.

## Bivalent and bidentate binding

Since dimeric hubs bind clients at two sites, these hubs can bind either one client chain containing two linear motifs or two client chains each containing one linear motif. We refer to these structures as bidentate (Fig. 1*C*) or bivalent (Fig. 1*D*), respectively. The bidentate binding mode results in high affinity binding for 14-3-3 and is therefore its common binding mode. LC8, however, binds exclusively in the bivalent mode. For hubs binding in a bivalent structure, either two copies of the same client chain can occupy both binding sites or two different chains can occupy each binding site. In cases where a bivalent hub binds to two different client chains, we refer to the function of the hub in this interaction as a bivalent adaptor. The presence of two binding sites in dimeric hubs makes these proteins structurally well-suited as adaptors as has been proposed for both 14-3-3 (24) and LC8 (25). The bivalent binding mode differs from bivalent adaptors in that in the bivalent mode, two copies of the same client are bound.

### Dimeric hub proteins 14-3-3 and LC8 play essential roles in eukaryotic cells

14-3-3 proteins are dimeric phosphoserine/phosphothreonine binding proteins (26) that regulate signaling, G2/M checkpoint release (27, 28), apoptosis (29, 30, 31), and transcription (32). Gene ontology analysis suggests over 1200 binding partners of 14-3-3 (33) in a wide range of cellular pathways (34, 35, 36). The effect of 14-3-3 interactions can broadly be placed in four categories: 1) prevention of dephosphorylation/degradation (37), 2) regulation of enzymatic activity (38), 3) alteration in subcellular localization by blocking a nuclear localization/exclusion sequence (39), and 4) chaperone-like function (40).

**Figure 2:**
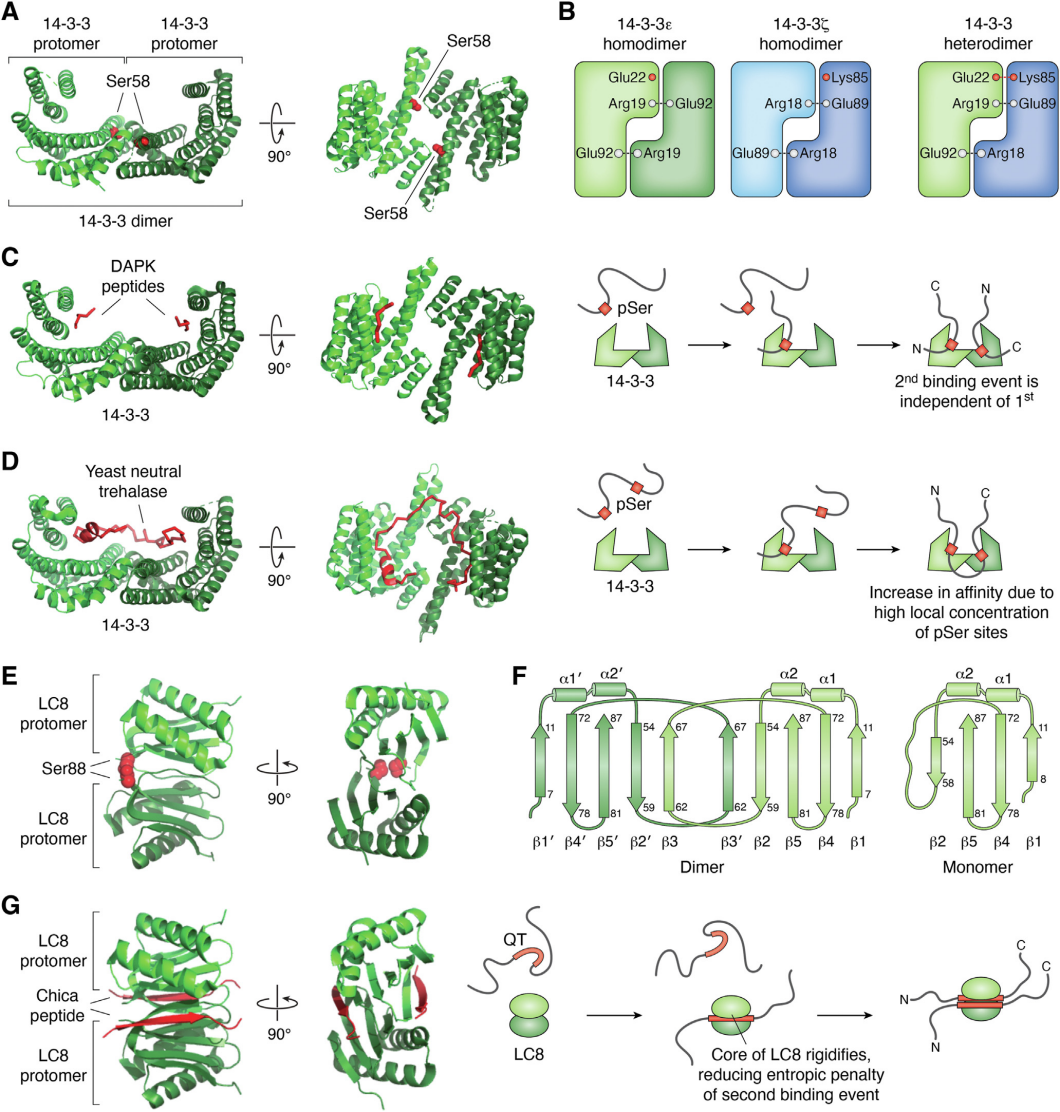
**Structures and interactions of dimeric hub proteins 14-3-3 and LC8.***A*, structure of apo-14-3-3 showing each protomer in a different shade of *green* and phosphorylation site Ser 58 in *red*. *B*, a diagram showing dimerization of 14-3-3 isoforms. Heterodimers can be stabilized by salt bridges as shown for heterodimers between 14-3-3 ε and ζ isoforms. *C*, structure of 14-3-3 in complex with two peptides of DAPK2. *D*, structure of 14-3-3 in complex with yeast neutral trehalase. *E*, structure of apo-LC8 (76) with each protomer shown in a different shade of *green* and phosphorylation site Ser88 side chain highlighted in *red*. *F*, Structural topology of LC8 dimer and monomer. Dimerization is accompanied by rigidification of β3, which forms a β-sheet when interacting with β2′ in the other strand in the dimer. *G*, LC8 bound to Chica peptide (51). The LC8 binding site shifts to accommodate the client chains and both the client and β3 of LC8 adopt a β-sheet conformation.

14-3-3 proteins are composed of two ∼30 kDa monomers each forming a cup-shaped structure of nine antiparallel alpha-helices containing one phosphopeptide binding site (41, 42, 43) (Fig. 2*A*). The antiparallel dimer contains a large central groove that can accommodate two client strands in an antiparallel orientation (44). The two binding pockets result in 14-3-3 binding either two client strands (Fig. 2*C*) or a single strand in a bidentate binding mode (Fig. 2*D*). 14-3-3 can also form various heterodimers composed of mixtures of isoforms (45) mediated by a series of inter-dimer salt bridges (Fig. 2*B*). Some isoforms preferentially form heterodimers which are stabilized by additional salt bridges not formed in homodimers (46). Clients of 14-3-3 may show isoform-specific binding preferences (47, 48, 49). This formation of heterodimers of 14-3-3 and binding preferences displayed by clients for specific 14-3-3 isoforms led to the hypothesis that 14-3-3 can act as a bivalent adaptor protein, linking together two different client proteins (45).

LC8 is a dimer composed of two ∼10 kDa monomers each binds disordered regions of clients and promotes their dimerization (50, 51, 52). LC8 was named for its initial discovery as a light chain subunit of the axonemal dynein complex but later has been revealed to have a universal role as a dimerization hub (1, 50), binding over 100 clients with a wide variety of functions and regulating them through dimerization of the client strands. Clients of LC8 are involved in various functions including regulation of dynein assembly (53, 54), DNA repair (55, 56), and transcription (57, 58). LC8 is expressed throughout the cell cycle and broadly distributed in cells (59), with fractions found in the cytoplasm, nucleus (56), membrane-bound complexes (60), and phase-separated condensates (61). LC8 is an essential gene, and knockouts of LC8 are not viable in *Drosophila* and mice (62, 63).

The LC8 dimer has two beta-sheets forming the dimer interface and a pair of alpha-helices surrounding this beta-sheet (Fig. 2*E*) (64). In its monomeric form, β3 is unstructured but is stabilized by interaction with β2 from the other protomer which forms the two symmetric binding grooves (Fig. 2*F*) (65). The disordered region in the client sequence adopts a beta-sheet conformation along the binding interface, with the client strand from each chain binding in a parallel configuration (64, 66) (Fig. 2*G*). In addition to rigidification and folding of the client strand upon binding, LC8 itself undergoes a structural change in which a shear opening of the binding groove increases the hydrophobic binding area to accommodate the client strand within the binding groove. Due to its dimeric structure, it was proposed that LC8 functions as a cargo adaptor where LC8 would bind dynein IC with one binding site, and the cargo with the other. To date, all structures of LC8-containing complexes have two copies of the same client binding both LC8 binding sites (1, 50, 67) arguing against the cargo adaptor hypothesis and in favor of our dimerization hub function. However, the Bim/Bmf complex involved in mitochondrial apoptosis forms a complex in which LC8 plays a role as a bivalent adaptor (25). The study used a short peptide of Bmf containing the LC8 recognition motif. Since this peptide coimmunoprecipitated with Bim, even though it did not contain the Bim binding sites, it provided strong support for LC8 as a bivalent adaptor binding both Bim and Bmf, in addition to its well-established function as a dimerization hub. Table 1 lists the binding modes and functions of different clients for LC8 and 14-3-3 which will be discussed in some detail in this review.

**Table 1:** Binding modes and functions of dimeric hub interactions

Hub	Client	Binding mode	Function
LC8	Swallow (93)	Bivalent	Coiled-coil stabilization
LC8	Dynein intermediate chain (IC) (53)	Bivalent	Bivalent scaffold, stabilization of other bivalent interactions (TcTex)
LC8	Bim/Bmf (25)	Bivalent adaptor	Stabilize interaction between Bim and Bmf
LC8	RavP (99)	Bivalent	Restrict conformational ensemble
LC8	ASCIZ (58, 77)	Bivalent 7–11 binding sites	Rheostat-like regulation through formation of ensemble of complexes
LC8	Nucleoporin 159 (Nup159) (60, 137)	Bivalent 5 binding sites	Rigidification of dynamic protein complexes
LC8	p53 binding protein 1 (53BP1) (141, 143)	Bivalent bridging 3 binding sites	Heterogeneous higher-order oligomerization
LC8	Leber congenital amaurosis 5 (LCA5) (148)	Bivalent bridging 2 binding sites	Higher-order oligomerization
14-3-3	SARS Cov2 N (101, 105)	Bivalent/Bidentate	Not known
14-3-3	Cystic Fibrosis Transmembrane Conductance Regulator (CFTR)	Bidentate	CFTR folding, protection from degradation, localization
14-3-3	Death-associated protein kinase 2 (DAPK2) (37)	Bivalent	Stabilize autoinhibited structures
14-3-3	Tau (122)	Bivalent/Bidentate	Stabilize/destabilize aggregates

### Bidentate 14-3-3 interactions

14-3-3 recognizes phosphoserine or phosphothreonine-containing motifs that are often located within disordered regions of client peptides (33, 68). Since many clients of 14-3-3 are phosphorylated at multiple sites, 14-3-3 can participate in bidentate interactions by binding two phosphopeptide motifs on the same client. In its interaction with aminopeptidase N (APN), the affinity of doubly phosphorylated constructs (K_d_ = 0.0046 μM) is increased over the singly phosphorylated construct by a factor of 500 (K_d_ = 24 μM) (69). Isothermal titration calorimetry shows that while there is a significant entropic penalty due to the conformational restraints imposed by 14-3-3 binding, the enthalpic contribution from bidentate binding significantly overcomes the entropic penalty. Due to the antiparallel dimer formed by 14-3-3, this binding mode forces a turn in the client peptide, which can result in a reduction of the distance between domains in the termini of the protein. This type of structure is seen in the activation of *Saccharomyces cerevisiae* neutral trehalase (Nth1) by 14-3-3 (38, 70). The binding of 14-3-3 stabilizes the regulatory Ca^2+^-binding domain of *N*th1, orienting the regulatory domain near the catalytic domain.

The affinity of bidentate 14-3-3 interactions is governed not only by the sum of the affinities for a single site but also by the structure and length of the linker separating them. A combination of fluorescence polarization, isothermal titration calorimetry, and microscale thermophoresis shows both enthalpic and entropic differences in the binding of 14-3-3 peptides to LRRK2 and CFTR due to differences in recognition motifs specificity and to the ordering of the linker between phosphopeptide motifs (71). In general, the free energy of binding for a doubly phosphorylated construct is close to the sum of the free energies of the singly phosphorylated constructs, suggesting minimal enthalpic contributions from the linker. However, for a doubly phosphorylated construct binding enthalpy is considerably increased compared to the sum of the two singly phosphorylated binding enthalpies. This increase is attributed to an extended binding interface as revealed by the crystal structure which includes residues outside the 14-3-3 binding site usually considered part of the linker (71, 72).

### Bivalent LC8 interactions

LC8 binds to intrinsically disordered regions of proteins containing short linear LC8 recognition motifs. The core of this motif is a three-residue long stretch most often a TQT or structurally similar sequence (IQT or VQT) referred to as the anchor sequence. While the entire sequence is variable, the anchor shows the least variation, and mutations in the anchor have the most destabilizing effects on LC8 binding.

Crystal structures of LC8 complexes all contain one dimer of LC8 and two copies of identical client strands occupying its binding grooves (73, 74, 75, 76). Upon binding a client strand, LC8 undergoes a conformational change which increases the hydrophobic surface available to bind as well as rigidifies the LC8 core (76). The rigidification in the LC8 pocket caused by the binding of the first chain pays the entropic cost and favors the binding of the second chain. NMR (76) and mass spectrometry (77, 78) provide clear evidence for a singly-bound client, but these are only seen at the low concentrations needed for mass spectroscopy or with sub-stoichiometric quantities of client peptide in NMR. Bayesian analysis of ITC data has revealed positive cooperativity in the two-step binding mechanism of LC8 (79) supporting the hypothesis that rigidification of the core of LC8 pays the entropic cost of the second binding step.

### Bivalent adaptors

Bcl-2 family proteins are important regulators of apoptosis. In particular, proapoptotic Bcl-2 homology three-only (BH3) proteins such as Bim and Bmf play a role in Bax/Bak-mediated cytochrome c release leading to mitochondrial apoptosis (80). The interaction between Bim and LC8 inhibits the proapoptotic activity of Bim (81, 82, 83). Starvation conditions result in phosphorylation of Bim, subsequent dissociation of Bim and LC8, and activation of Bim proapoptotic activity (82). Due to the dimeric structure of LC8 and its canonical role in the dynein complex, it was proposed that LC8 links Bim to the dynein motor complex to sequester it from diffusing in the cytoplasm (81). However, more recently it has been shown that Bmf binds directly to both LC8 and Bim (25). A ternary complex in which a single dimer of LC8 binds to both Bim and Bmf is supported by a pulldown assay in which short peptides of Bmf simultaneously interact with LC8 and Bim. The short LC8 binding peptides used in this experiment did not include the heterodimerization domains of Bim and Bmf and their simultaneous binding provides support for the formation of an LC8 complex with two different clients. Although this is the only reported observation of a complex with two different clients, future work will determine whether such LC8 assemblies that bridge two different clients are observed in other systems.

### Phosphorylation of both hub and client regulates interactions

14-3-3 recognizes several variable sequences that contain a phosphoserine/threonine, making phosphorylation an important determinant of 14-3-3 binding (84), but several noncanonical clients bind 14-3-3 in the absence of phosphorylation (85, 86). Phosphorylation of 14-3-3 at Ser58 results in monomerization of 14-3-3 (Ser58 highlighted in red in Fig. 2*A*) (87). Recent advances in genetic code expansion have allowed the production of constitutively phosphorylated 14-3-3, and pulldown assays using this construct suggest that the interactome of 14-3-3 is significantly altered upon phosphorylation (88).

LC8 is also regulated through both hub and client phosphorylation. Phosphorylation of LC8 clients at or near the anchor sequence results in a dramatic reduction of binding affinity (75, 89). Additionally, LC8 can have its dimerization destabilized by phosphorylation. Phosphomimetics of LC8 at Ser88 close to the dimer interface of LC8 resulted in dimer dissociation (Ser88 highlighted in red in Fig. 2*E*) (90) and dramatically decreased affinity to clients. However, NMR experiments show that binding was restored at high concentrations for the higher affinity clients, suggesting that a small population of dimeric LC8 binds to clients, stabilizing the LC8 dimer and resulting in a significant population of dimerized LC8. The role of LC8 phosphorylation is therefore to select for higher affinity clients.

## Polybivalency

### Swallow formation of a bivalent scaffold

**Figure 3:**
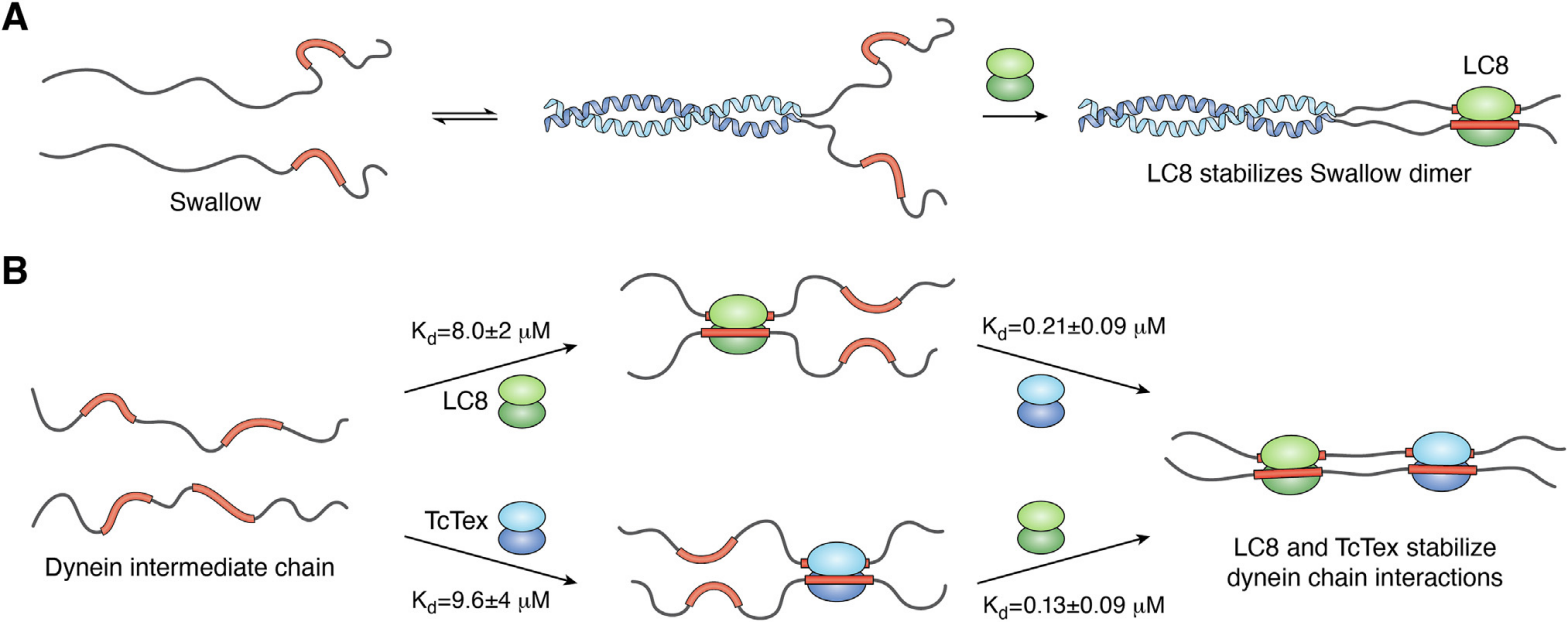
**Polybivalent binding.***A*, with Swallow (Swa), LC8 binds to a subpopulation of dimerized Swa, stabilizing the dimeric form. *B*, in the dynein intermediate chain, binding of multiple dimeric light chains such as TcTex and LC8 greatly stabilizes the interaction (53).

LC8 binding induces a clear disorder to β-strand transition at the binding interface, and structural changes distant from binding. For example, Swallow, involved in the localization of RNA in *Drosophila* oocytes (91, 92), contains a coiled-coil 15 residues N-terminal of the LC8 binding motif (Fig. 3*A*). The self-association of the coiled-coil in Swallow is weak, but its presence increases the affinity of LC8 binding indicating energetic coupling between LC8 binding and coiled-coil formation. Mutations of the coiled-coil that create monomeric Swallow decrease LC8 affinity from 200 nM to 500 nM, while a variant that forms a constitutive dimer increases affinity to 70 nM (93). The outcome is that LC8 stabilizes the dimerized complex, resulting in a larger population of dimerized Swallow. This 7-fold increase in affinity is caused by the formation of a bivalent duplex in Swallow caused by self-association. Coiled-coils and other self-association domains are commonly found near LC8 binding sites, and similar stabilization of weak self-association domains is also seen in the dynein intermediate chain (IC) (94).

### The dynein intermediate chain (IC)

Another example of a polybivalent effect in LC8 binding is its interaction with dynein IC. IC binds TcTex, another dimeric light chain that is a structural homolog of LC8 (Fig. 3*B*). When titrated into a preformed complex of IC and TcTex, the affinity of LC8 for IC increases from 8 μM to 0.13 μM, well over a 50-fold increase. IC also binds cargo adaptors NudE and dynactin p150 at its N-terminal region (95).

Polybivalent interactions are involved in regulating the activity and cargo binding of IC (54). IC forms a compact, autoinhibited state in which an N-terminal α-helical region binds to a light chain LC7 binding site more than 150 amino acids away in sequence. This compact structure covers the N-terminal α-helical region which is the binding site to at least two dynein regulators and cargo adaptors, resulting in reduced affinity of IC for dimeric dynactin p150, but completely abolishing binding to NudE (54). Introducing LC7 which will bind to the LC7 binding site, creates a polybivalent scaffold, opens the autoinhibited structure, and makes IC accessible for binding p150 and NudE.

### LC8 and rabies phosphoprotein (Rav P)

**Figure 4:**
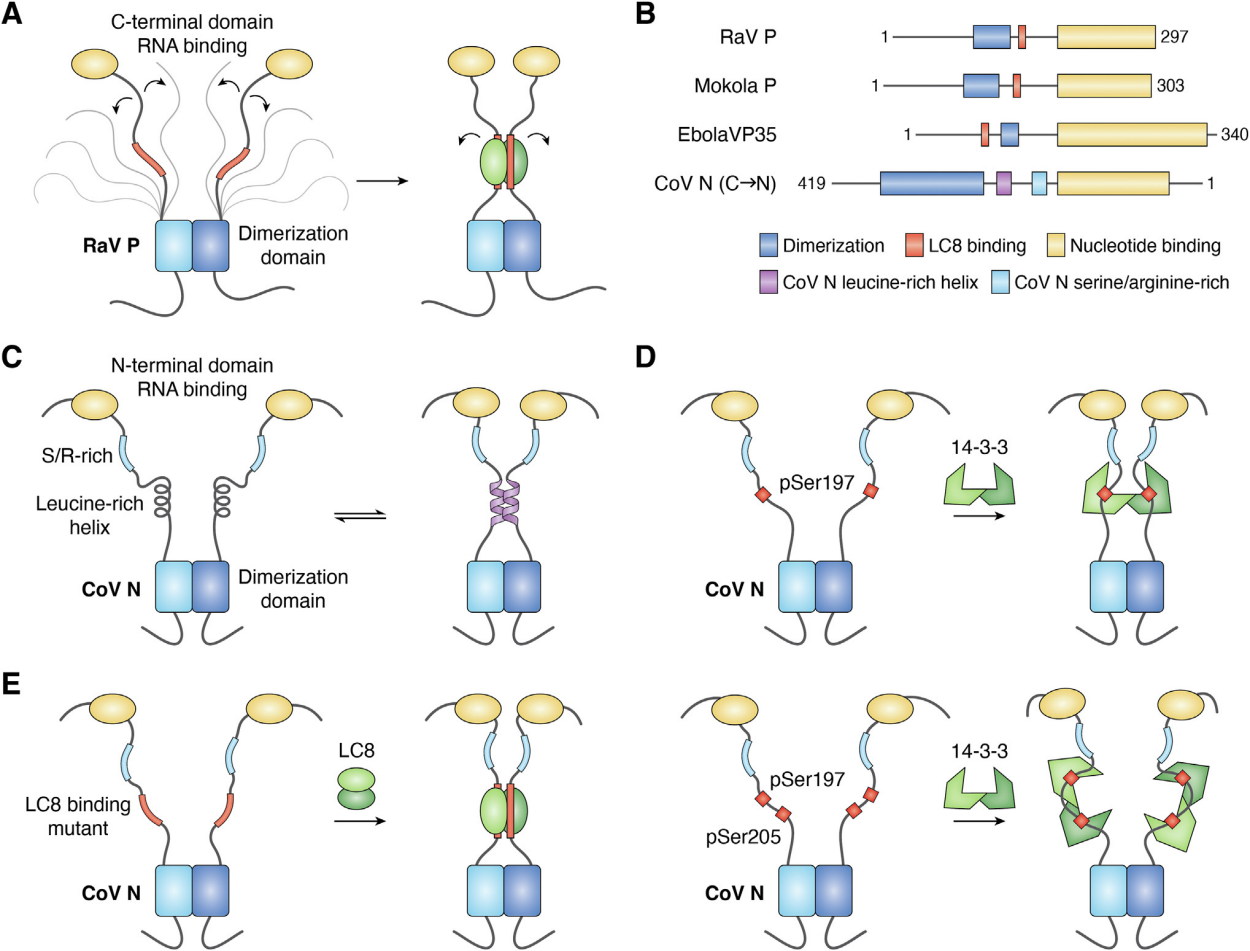
**Viruses hijack dimeric hubs to improve interactions with nucleic acids.***A*, Rav P has a dimeric N-terminal domain. Binding to LC8 elongates the region of dimerization in Rav P, which restricts the conformational ensemble of the complex, resulting in the nucleotide-binding C-terminal domains remaining near each other. *B*, the domain architecture of selected viral proteins. In each, the distance between dimerization and nucleotide binding is conserved. *C*, Cov N RNA binding and phase separation are dependent on transient self-association of the leucine-rich helix. *D*, binding of 14-3-3 to phosphorylated forms of Cov N may dimerize the N linker near the leucine-rich helix, which would impact RNA binding and phase separation, or cover the phosphorylation site in both chains. *E*, a variant of Cov N which incorporates an LC8 binding site in place of the leucine-rich helix rescues phase separation in the presence of LC8.

While rabies is completely lethal in mice, removing LC8 binding by mutation of rabies phosphoprotein (Rav P) results in a nearly nonlethal viral infection (96). Rav P contains a dimerization domain (97) ∼15 residues N-terminal from an LC8 binding site (98) (Fig. 4*A*). While Rav P is strongly dimerized even in the absence of LC8 binding, NMR and SAXS data show that LC8 binding restricts the structural ensemble of Rav P by aligning the C-terminal domains, thereby reducing conformational heterogeneity of Rav P, which is proposed to facilitate transcriptional activity (99). Binding to LC8 is therefore thought of as a switch that induces a more active conformation. LC8 binding is also reported in both human parainfluenza and Ebola virus phosphoproteins (100), which contain tetrameric coiled-coil domains.

### 14-3-3 and SARS Cov-2 nucleocapsid protein (N)

SARS Cov-2 nucleocapsid protein (N) is a dimeric protein that binds RNA at multivalent sites (101). It contains a C-terminal dimerization domain (CTD) (102) and an RNA-binding N-terminal domain (NTD) (103). Between these domains is a serine/arginine-rich region (SR-rich) (residues 176–206) which can be multiply phosphorylated to regulate higher-order oligomerization and phase separation (104) (Fig. 4*C*). It also contains a leucine-rich helix (LRH) (residues 218–231) that is prone to self-association (101). Each protomer of 14-3-3 binds to one phosphopeptide in N, creating a 2:2 complex that has similarities with the Rav P: LC8 complex (Fig. 4*D*) (105, 106, 107). Both complexes bind to a bivalent scaffold formed by the viral client, bringing together disordered regions of their viral client. One proposed function of the interaction of 14-3-3 is to protect the binding sites from dephosphorylation (107), which is expected to reduce the phase separation of N^75^. Interestingly, a chimeric variant of N containing an LC8 binding site in the linker region replacing the LRH rescues phase separation (Fig. 4*E*) (101). While phosphorylation of N downregulates phase separation and 14-3-3 may protect these sites from dephosphorylation, it is possible that 14-3-3 mediated self-association could also result in self-association of N and stimulate its phase separation in addition to protection from phosphorylation.

### Potential conserved role of dimeric hubs in viral hijacking

The role of LC8 binding to Rav P appears to be extending its dimerization region, which will restrict the conformational ensemble of Rav P and aid in orienting the nucleotide-binding domains. The vesicular stomatitis livestock virus phosphoprotein (VSVP) does not have an LC8 binding site but has a longer dimerization domain in comparison to Rav P^67^. The result is that the linkers separating the dimerization domain and nucleotide-binding domains in both Rav P and VSVP are of similar length. SARS CoV2 N protein has an SR-rich region near the LRH which undergoes transient self-association (101). Replacing the LRH with an LC8 binding site results in robust phase separation of N in the presence of LC8 and RNA, suggesting that extending the region in which N dimerizes improves phase separation due to improved interactions with RNA. This supports the hypothesis that restriction of disorder in the linker region either by LC8 binding or by self-association regulates phase separation and that the LRH in SARS CoV2 N has a similar role as LC8 binding in Rav P. Interestingly, the several sites in or near the LRH (S197 and S205) can be phosphorylated and bind 14-3-3 (105, 106) making it possible for 14-3-3 binding to stabilize self-association by bivalent interactions.

## Heterogeneity of dimeric hub protein complexes

### Compositional and conformational heterogeneity

Dimeric hub proteins often form complexes containing multiple interconverting subpopulations with varying stoichiometry (compositional heterogeneity) or structures (conformational heterogeneity) under equilibrium conditions. Compositional heterogeneity is defined as the variation in stoichiometry and binding energetics within a population of multivalent protein assemblies, while conformational heterogeneity is characterized by the dynamic interconversion of multiple conformations within a molecular population (108). Here we explore structural causes and functional outcomes of heterogeneity in these hub proteins.

### Isoform heterogeneity of 14-3-3 in the cell

While 14-3-3 proteins are highly conserved eukaryotic proteins, the number of their isoforms can differ from species to species. In *Drosophila*, there are only two, while plants express up to ten (45) and humans express seven (109). Different isoforms show different propensity for forming homo-or heterodimers (45). For example, human 14-3-3σ forms almost exclusively homodimers, whereas 14-3-3β forms a much higher proportion of heterodimers (49, 110). Dimerization of 14-3-3 is driven by inter-protomer salt bridges in the first four helices which show higher sequence diversity between isoforms than other parts of the sequence. Some isoforms may have specific functions not shared with others, such as the role of 14-3-3σ in DNA repair. Some heterodimers bind to multiple clients simultaneously, possibly acting as a scaffold to bridge the two clients (111, 112). 14-3-3 isoforms therefore increase heterogeneity of 14-3-3 complexes with diverse functions.

### Heterogeneity of polyphosphorylated clients

**Figure 5:**
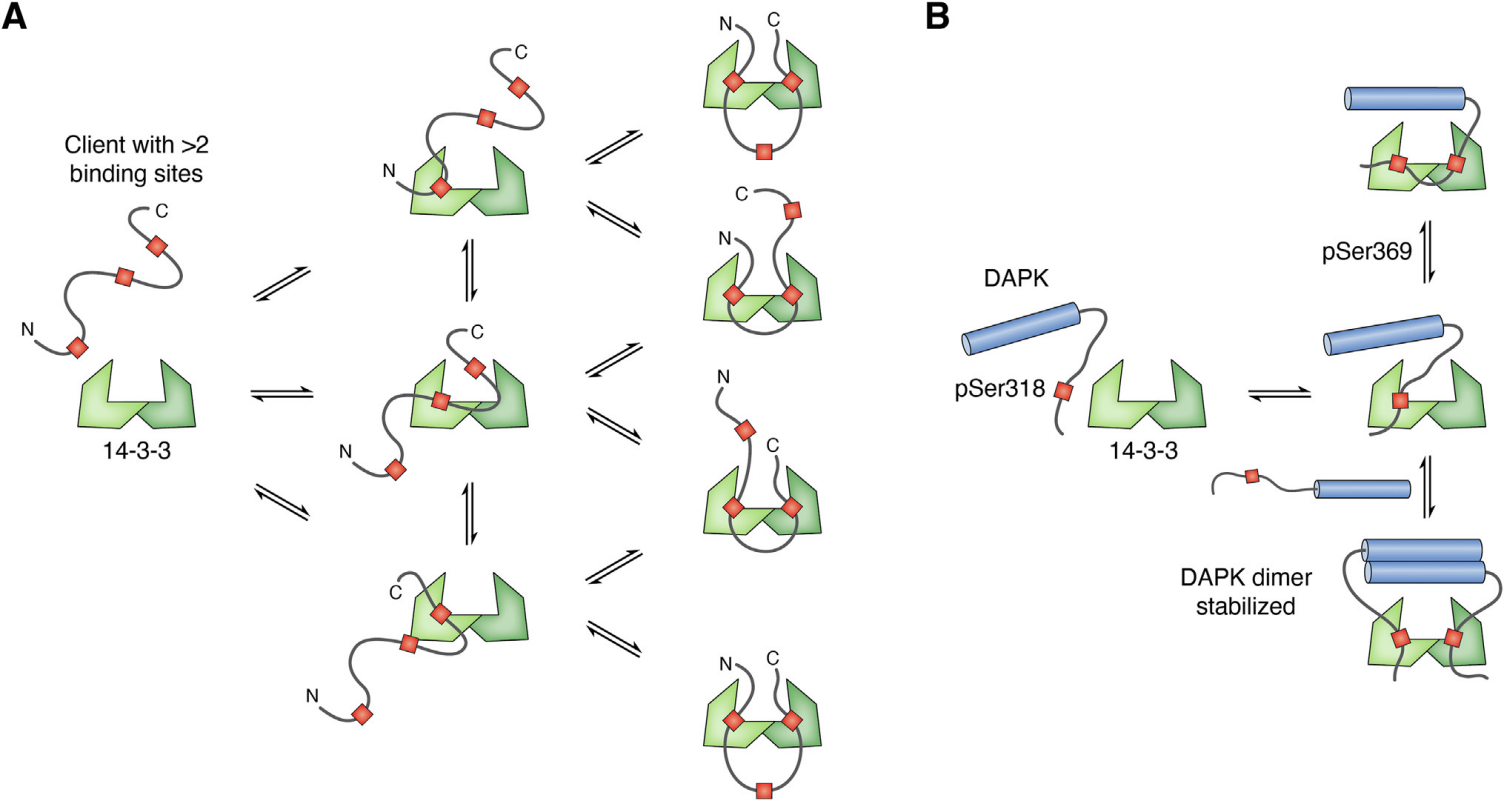
**Mechanisms leading to heterogeneity in 14-3-3 complexes.***A*, a 14-3-3 client with more than two binding sites forms a mixture of complexes with different occupancy. *B*, in its interaction with DAPK2, 14-3-3 stabilizes the autoinhibition of DAPK2 by promoting the formation of dimers and protecting the phosphorylation in disordered regions of DAPK2.

The multiple phosphorylation sites in a client can result in compositional heterogeneity of the complex (Fig. 5*A*). Three or more 14-3-3 recognition sites in a client introduce the possibility for the formation of multiple high-affinity complexes. In CFTR, a triply phosphorylated construct shows a higher affinity (7.8 μM) for 14-3-3 than a doubly phosphorylated construct (13 μM) (71). The highest affinity site, pS768, likely binds first and then is stabilized by contacts with either pS753 or pS795. The singly and doubly bound states result in structural heterogeneity since bidentate binding of 14-3-3 requires a structural change in the client while the singly bound complex does not. In clients with more than two or more phosphorylation sites, multiple copies of 14-3-3 could bind to a single client chain (23). For example, there are between three and seven 14-3-3 binding sites in Tau (113, 114), six sites in LRRK2 (71, 72), and nine in CFTR (39, 115). In such interactions, there is often a dominant bidentate interaction, but thermodynamic modeling of CFTR and LRRK2 suggests the formation of a mixture of complexes bound at different sites, further contributing to the heterogeneity of 14-3-3 complexes (23, 71).

### Multiple binding modes of 14-3-3

In addition to the cooperative binding mode in which 14-3-3 binds to two sites on a single chain of client, 14-3-3 can bind singly phosphorylated client strands. In singly phosphorylated clients, 14-3-3 forms a stable and homogeneous complex with the client chains oriented in an antiparallel orientation, in contrast to the parallel dimers formed by LC8 (116, 117).

In doubly phosphorylated clients that can form dimers, a mixture of complexes with 1:2 and 2:2 stoichiometry may be formed (37). Death-associated protein kinase 2 (DAPK2) is a kinase involved in apoptosis, autophagy, granulocyte differentiation, and motility regulation, and DAPK2 activity is regulated through autoinhibition. DAPK2 is autoinhibited in its dimeric form (118), which is regulated through phosphorylation as Ser369 and Ser318 (37, 119). A titration of 14-3-3 into DAPK2 shows a slow shift from a peak with a low sedimentation value (Svedberg, S) to a second peak with a higher S value (37), suggesting an exchange between the two forms. The addition of 14-3-3 results in a larger proportion of dimeric complex. DAPK2 binding to 14-3-3 in a 2:2 stoichiometry may serve as a mechanism for the regulation of DAPK2 function. The singly bound complex can exchange between bidentate and bivalent binding modes, resulting in a change in the dimerization of DAPK2 and regulating its activity through stabilizing autoinhibited states.

### Heterogeneity in 14-3-3 interactions with Tau

Tau, originally identified for its function in microtubule assembly (120), is an intrinsically disordered protein that partially adopts a closed structure in which the N- and C-termini fold close to central microtubule-binding domains (121). Dysregulation of Tau can result in aggregation and formation of amyloid plaques, which are associated with neurodegenerative disorder (122). The phosphorylation state of Tau is complex and features many sites that can be phosphorylated, and phosphorylation of these sites alters the affinity of 14-3-3 interactions (113, 123). 14-3-3 can bind one (124) or two (125) Tau strands, which may contribute to the stabilization of the compact form of Tau. The binding of 14-3-3 to Tau regulates its function by competing for binding to tubulin and regulating the phosphorylation of Tau (124, 126). 14-3-3 binding reduces the formation of liquid condensates on microtubules (127), which appear to reduce amyloid formation.

14-3-3 promotes the aggregation of nonphosphorylated Tau (128) and can be found within these aggregates (129). This complex would contain 14-3-3 binding in the bivalent mode, with each site bound to a different strand of Tau, which stabilizes intermolecular interactions resulting in amyloid formation. Phosphorylation of Tau reduces aggregation in the presence of 14-3-3 *in vitro* (128) bound in a bidentate complex (122). The stabilization of the Tau structure through bidentate contact with 14-3-3 may explain the loss of aggregation in phosphorylated Tau constructs. The network formed by 14-3-3 binding heterogeneously to Tau (or other proteins capable of phase separation) could in principle result in higher-order assemblies that form a network like those formed in liquid condensates.

### Heterogeneity in LC8 polybivalent assemblies

Clients of LC8 with multiple recognition motifs bind LC8 in a polybivalent assembly or form a multivalent LC8 complex (130). A variant of IC in which the TcTex binding site is replaced by a second LC8 binding site has a 1000-fold binding improvement for the binding of the second LC8 dimer (53). In such complexes, LC8 binds its client at multiple sites within the client chain and dimerizes the client generating a symmetric complex. As many as 11 binding sites have been found in the multivalent LC8 binding client, ASCIZ.

While there is some evidence of off-register binding and complexes containing only a single client strand, the thermodynamic enhancement from polybivalency results in almost completely in-register complexes that contain two chains of the same client (78). The preference for in-register complexes may be explained by the binding improvement from a bivalent scaffold. If one considers a client of LC8 with two LC8 binding sites, like the synthetic two-site IC construct described earlier in this section, the in-register configuration forms a bivalent scaffold in which two LC8 sites are brought into proximity with each other, increasing the effective molarity of these sites. The off-register complex, in contrast, would move the unbound LC8 sites far from each other resulting in no binding improvement expected from polybivalency.

### ASCIZ/LC8 interactions form a rheostat-like assembly that regulates transcription

**Figure 6:**
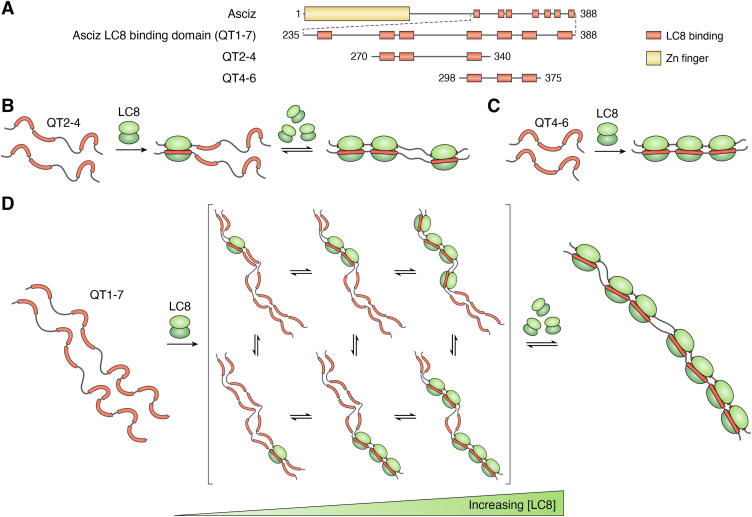
**Multivalent clients of LC8 form polybivalent assemblies.***A*, domain maps of ASCIZ from *Drosophila melanogaster*. ASCIZ contains 7 LC8 binding sites in this species. Domain maps for QT2-4 and QT4-6 are also included. *B*, QT2-4 of ASCIZ binds in a stepwise manner, filling QT2 first. When fully bound, it exhibits conformational heterogeneity, with QT2 and QT3 showing different dynamic motions than QT4 in the complex. *C*, QT4-6 binds cooperatively, with all sites filling in a single binding step. *D*, titrating the full LC8 binding domain of ASCIZ, QT1-7, gives a heterogeneous mixture of complexes with different stoichiometries.

The ATM substrate Chk2-interacting Zn2+ finger protein (ASCIZ, or ATMIN) is a transcription factor involved in the DNA damage response (131) which has its subcellular localization regulated by interactions with LC8 (57). Interestingly, ASCIZ is the transcription factor for LC8. ASCIZ binds LC8 multivalently, with up to 11 LC8 binding sites depending on the species (11 in humans, seven in *Drosophila*) (Fig. 6*A*). The complex formed between ASCIZ and LC8 is heterogeneous as shown by analytical ultracentrifugation, with a low LC8 occupancy intermediate that remains partially occupied even in high excess of LC8 (Fig. 6*D*) (58). The level of occupancy correlates with the level of transcription, with variants that bind less LC8 generally showing higher transcription (58). ASCIZ therefore functions as a concentration sensor for LC8 such that when ASCIZ has low LC8 occupancy, there is less inhibition of transcription, which results in increased transcription of LC8. The reverse is true for high LC8 occupancy.

The ability of ASCIZ to act as a concentration sensor for LC8 can be explained by cooperative binding in two different regions within ASCIZ. The full LC8 binding domain of ASCIZ forms heterogenous complexes at all concentrations, but shorter ASCIZ constructs (QT1-3 and QT4-6) show independent peak attenuation by NMR (58), simpler titration behavior analyzed by AUC, and form homogeneous complexes when bound to LC8. While QT4-6, a construct containing sites 4, 5, and 6, binds cooperatively, the low mass and broad peaks in the QT2-4, a construct containing sites 2, 3, and 4, suggests that complexes in exchange may be formed in sub-saturating quantities of LC8 (77), which may be due to the long linker separating QT3 and QT4. In addition, NMR dynamics of the QT2-4:LC8 complex show that QT2 and QT3 have similar dynamic behavior, while QT4 separated by a longer linker has lower R_2_/R_1_ values, which serves as a measurement of local dynamic motion. The similar R_2_/R_1_ values in QT2 and QT3 suggest that they tumble together, while a reduced R_2_/R_1_ for QT4 shows that it diffuses independent of QT2 and QT3. Together, these data suggest that the long linker separating QT3 and QT4 causes the formation of compositionally heterogenous ASCIZ:LC8 complexes (Fig. 6, *B* and *C*).

### Nup159 forms a rigid rod-like assembly through polybivalent interaction with LC8

The nuclear pore complex (NPC) is a large multiprotein complex that regulates nucleocytoplasmic trafficking of macromolecules (132, 133). Components of the nuclear pore complex are called nucleoporins (Nups) (134). Nucleoporin 159 (Nup159) is an important component of the NPC; however, the role of LC8 binding in Nup159 is currently not known. Nup159 contains five binding sites for LC8 between a Phenylalanine-Glycine (FG) repeat domain and a coiled-coil. LC8 is incorporated into the NPC late in assembly and may be incorporated in a stepwise mechanism (135). In support of stepwise binding of LC8 to Nup159, a construct containing the first 3 LC8 binding sites is more stable than a construct containing all 5^71^. This suggests that Nup159 may bind different numbers of LC8 dimers in a concentration-dependent manner. Negative stain electron microscopy of the complexes containing Nup159 and LC8 shows the LC8-binding domain (LBD) as a series of beads-on-a-string appearing as a stiff rod (60, 136). A titration of Nup159 with LC8 analyzed by NMR shows peak attenuation in the entire LBD (137), providing support for the rigidification of the LBD in response to LC8 binding. The elongation of Nup159 is proposed to help orient the FG repeats in Nup159 into the central channel of the nuclear pore, where unstructured FG repeat proteins contribute to regulating nucleocytoplasmic transport. In lower concentrations of LC8, the LBD is more disordered. When local LC8 concentrations rise, the LBD extends, which could force the FG repeats in Nup159 into the channel of the nuclear pore complex (60).

## Heterogeneity in bridging subcomplexes to stimulate higher-order oligomerization

### 53BP1

**Figure 7:**
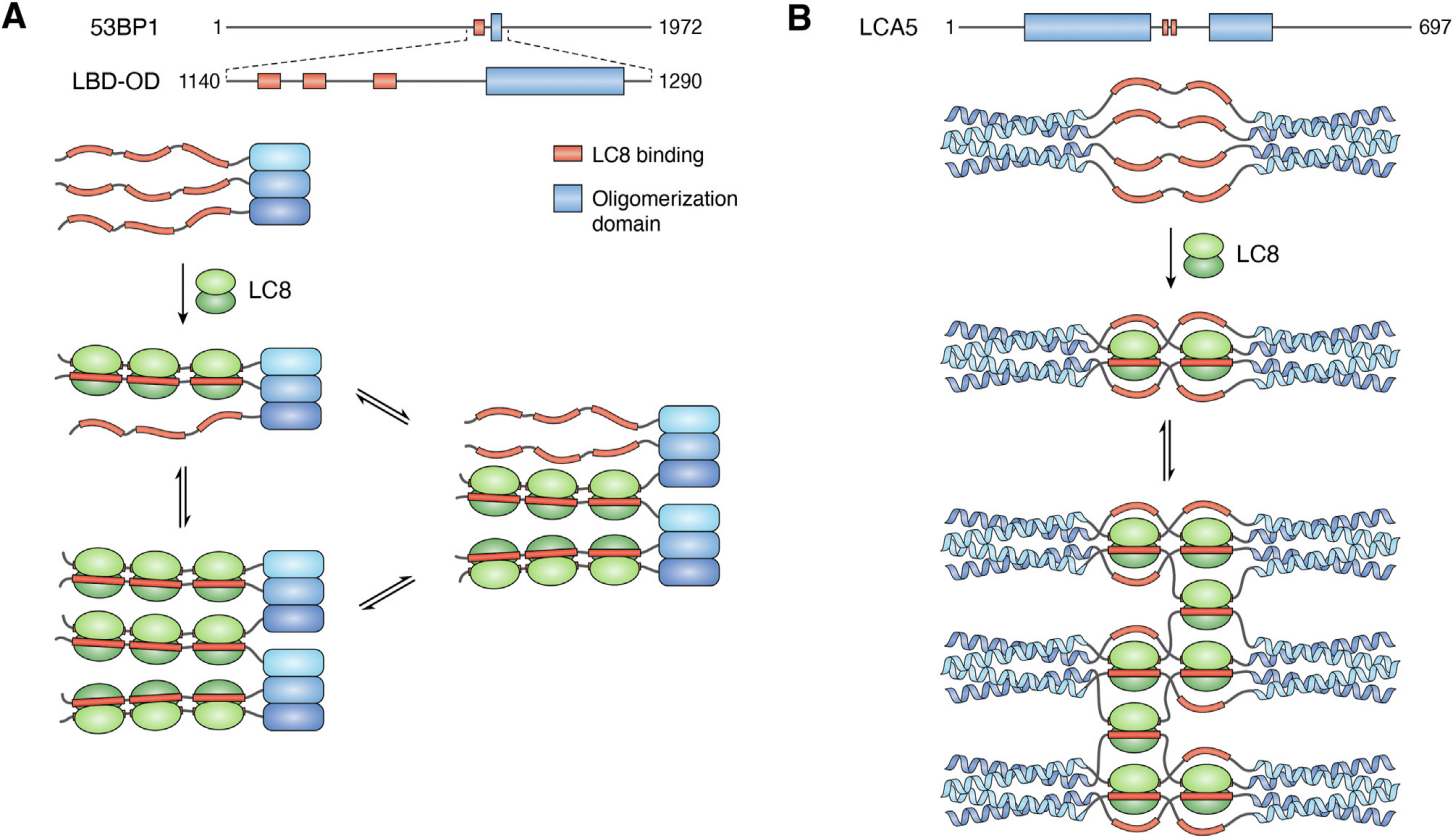
**LC8 bridging between subcomplexes of the client can produce heterogeneous complexes.***A*, 53BP1 is stably trimerized by its oligomerization domain (*blue* rectangles). Upon binding to LC8 dimer, 53BP1 forms a mixture of trimers and dimer-of-trimers. *B*, LCA5 contains two LC8 binding sites between its two tetrameric coiled-coils. Binding to LC8 results in the formation of larger assemblies bridged by LC8.

Tumor suppressor p53 binding protein 1 (53BP1) is a large scaffolding protein with functions in DNA repair and cell cycle control (138, 139, 140). 53BP1 binds LC8 (55, 141) at three sites (142) where LC8 is shown to regulate 53BP1 accumulation in DNA repair foci (55, 141) and the sensitivity of BRCA-negative cells to chemotherapy drugs (141). We have shown recently that 53BP1 contains a trimeric oligomerization domain ∼30 residues from an LC8 binding domain containing three binding sites. The binding of LC8 to the LC8 binding domain of 53BP1 (LBD) in the absence of the oligomerization domain is relatively simple, with a single population being formed as analyzed by size exclusion chromatography. The oligomerization domain (OD) is also homogeneous, forming completely trimeric populations in the concentration range of 7.5 to 75 μM (143). However, titration of 53BP1 LBD-OD results in a mixture of complexes with a mass between 120 and 350 kDa. This supports a model in which LC8 bridges two trimers of 53BP1 that exchange with a trimer resulting in a heterogeneous assembly (Fig. 7*A*). While 53BP1 contains 3 LC8 binding sites (red, Fig. 7*A*), only the middle site is necessary and sufficient for bridging the 53BP1 trimers. Variants that remove binding at this site do not form stable dimer-of-trimers complexes, forming instead only homogeneous trimer complexes. Focus formation assays in U2OS cells with this site mutated do not improve phase separation, suggesting that the formation of heterogeneous complexes with LC8 is necessary for 53BP1 DNA repair functions.

### LCA5

Lebercilin (Leber congenital amaurosis 5, LCA5) is a protein involved in the human ciliary-centriole system that is associated with the development of the disease Leber congenital amaurosis (144). The ciliary centrosome system contains many coiled-coil-forming proteins (145) with long regions of disorder (146). LCA5 has two coiled-coils (147) that surround two LC8 binding sites. Based on a 1:1 stoichiometry measure by ITC, the coiled-coil was inferred to be tetrameric. SEC profiles show that a species with a very large mass forms in a time-dependent manner, which appears as beads-on-a-string by electron microscopy. This suggests that LC8 bridges between tetramers of LCA5 form large, polymerized assemblies (Fig. 7*B*). The size of the complex is not limited by the number of LC8 sites available, as the addition of a new LCA5 tetramer to the assembly introduces another free LC8 binding site.

## Conclusion

### Cooperativity in binding

While both LC8 and 14-3-3 can bind clients at two binding sites to produce a high-affinity complex, the source of cooperativity is different for each hub. 14-3-3 is capable of binding clients at a single site, but the presence of a second site in some clients increases the local concentration of 14-3-3 sites resulting in cooperativity in 14-3-3 binding in the bidentate mode, with the overall affinity of the interaction being heavily influenced by the length of the linker separating the two 14-3-3 binding sites (71). Likewise, LC8 undergoes a structural rearrangement in its dimer interface upon binding a client at a single site which favors binding at the second site (76, 79), and is the reason that LC8 binds exclusively in the bivalent mode. With LC8 multivalent clients containing multiple binding sites, LC8 binding generates a polybivalent assembly that favors binding of LC8 at multiple sites with parallel orientation. The tendency of 14-3-3 to form high-affinity complexes in the bidentate mode does not generate such symmetric complexes, and the lack of proximity of 14-3-3 binding sites in multivalent clients of 14-3-3 may explain why 14-3-3 does not form polybivalent assemblies.

### Dimeric hubs and phase separation

While many clients of dimeric hubs undergo liquid-liquid phase separation, there are few examples clearly showing their involvement in the process. 14-3-3 reduces liquid condensate formation of Tau (127), and fusion of 14-3-3 binding sites to proteins that readily undergo phase separation both *in vivo* and *in vitro* reduces the number of foci generated (40). These experiments suggest a conserved role for 14-3-3 in reducing the phase separation by interfering with the multivalent interaction necessary for biological condensate formation (40). A similar role for LC8 was proposed in its interaction with human parainfluenza virus three phosphoproteins (HPIV3P) based on reduction in turbidity in the presence of LC8 (100). However, 53BP1 forms liquid condensates at sites of DNA damage which increase in number in the presence of LC8 binding, suggesting that there are cases in which binding bivalent hubs increase phase separation of clients (61, 141, 143). The increase in phase separation as a result of adding an LC8 binding site to the SARS Cov-2 N protein suggests that dimeric hubs may increase phase separation, as well (101).

### Features of LC8 clients that form heterogeneous complexes

Most structural research has focused on high affinity, homogeneous complexes which are a requirement for many high-resolution structural techniques such as X-ray crystallography. Heterogeneity complicates analysis even for techniques that can detect multiple conformations, such as NMR and Cryo-electron microscopy. It is well established that the binding of LC8 at disordered regions results in the dimerization of the client chains and ordered structure at the site of binding (50, 64, 66). Often, the binding of LC8 promotes the dimerization of self-associating domains (93, 130) or restricts the conformational ensemble of disordered regions of the client (99). In both functions, LC8 reduces the conformational heterogeneity of the client. Due to the cooperative binding mode of LC8 and the expected binding enhancement from the formation of a bivalent scaffold, one may expect complexes of LC8 to form exclusively homogeneous complexes. However, recent research has shown that heterogeneity may be an important feature of some polybivalent assemblies.

One cause of the heterogeneous binding of LC8 is the length of the linkers separating the multivalent LC8 sites. This is clearly observed in ASCIZ, in which QT2-4 and QT4-6 each containing 3 LC8 sites, both bind LC8 cooperatively but show distinct dynamic behavior (77, 78). Compositional heterogeneity with QT2-4 is clear from analytical ultracentrifugation, isothermal titration calorimetry, and multi-angle light scattering (58) while NMR shows conformational heterogeneity. In titration experiments, the peaks for the region containing QT4 through QT7 attenuate at lower concentrations of LC8 than the region containing QT1 through QT3, while relaxation experiments clearly show QT2 and QT3 behaving as a unit that is different from QT4. This suggests that cooperative effects in ASCIZ may be restricted to smaller regions within the LC8 binding domain and the formation of heterogeneous complexes is controlled by the length of the linkers separating them.

Another mechanism that contributes to heterogeneity is the bridging of client subcomplexes by LC8. In 53BP1, the binding of LC8 is stabilized by the formation of a pre-associated scaffold for only intra-trimer interactions, resulting in weaker inter-trimer (bridging) interactions. 53BP1 then exists in a mixture of trimer and dimer-of-trimer complexes which exchange depending on LC8 concentration. Since the dimer-of-trimer complex has all LC8 binding sites filled, LC8 cannot stimulate the oligomerization of a complex larger than the dimer-of-trimers. This results in a complex with an upper boundary to its oligomeric size. In contrast for LCA5 (148), bridging of tetrameric complexes results in daisy-chaining of LCA5 complexes that increase in average molecular weight over time. In this case, the upper limit of the complex size is not regulated by the number of LC8 sites in the complexes, and instead is likely determined by the kinetics of bridging and dissociation of LCA5 tetramers.

What stands out about these two mechanisms is that heterogeneity appears to be caused by multiple, distinct regions in which clients bind LC8, where in ASCIZ, there are multiple distinct binding regions in a single chain, while in addition, in 53BP1, there is an association of multiple chains containing multiple binding regions.

One may speculate that local concentrations of both client and hub protein and posttranslational modifications such as phosphorylation would regulate heterogeneity. An excellent example of such spatiotemporal regulation is seen with the interplay between LC8, and DNA repair proteins 53BP1 and MRE11. Interactions with 53BP1 localize LC8 to nuclear foci, and phosphorylation of LC8 results in dissociation from 53BP1 and interaction with MRE11 (56, 149).

### Dimeric hubs form unique complexes

The dimeric hub proteins, 14-3-3 and LC8, are ubiquitously expressed in eukaryotes and involved in diverse cellular functions. While 14-3-3 and LC8 can both bind clients at two sites, they do so with very different configurations and mechanisms. LC8 always binds to two client strands, forming a dimeric complex with a parallel configuration. 14-3-3 can form either a bivalent complex in which two client strands are bound in antiparallel or a bidentate complex which forces a turn into a single client strand. While LC8 always binds clients in a bivalent mode, the multiple ways of 14-3-3 binding can have opposing effects, for example, binding of 14-3-3 to Tau in a bivalent binding mode may promote pathogenic amyloid formation, while the bidentate mode reduces amyloids. The exclusivity of the structures formed by 14-3-3 and LC8 binding offers a reason for the evolution of multiple dimeric hubs. Therefore, while 14-3-3 and LC8 superficially appear similar in their dimeric structure and ability to bind a client peptide at two symmetric sites, there is little functional overlap due to the diversity of the bound structures of each hub.

The formation of polybivalent complexes is a common mechanism for dimeric hub proteins. The additional bivalent site(s) can stabilize a weakly dimerized client (Swa, DAPK2), restrict the conformational ensemble of strongly dimerized client (RavP, Nup159, SARS Cov-2 N), or contribute to compositional heterogeneity of client complexes (ASCIZ, LCA5, 53BP1, CFTR). However, differences in client chain orientation for 14-3-3 and LC8 in the bound form result in differences in the functions regularly played by each hub. The parallel binding of client chains by LC8 tends to elongate clients of LC8, while antiparallel binding by 14-3-3 generates more compact structures. The high affinity afforded by polybivalency in complexes containing LC8 results in stable complexes which often have increased affinity for downstream bivalent interactions. LC8 therefore functions as a part of complex assembly. 14-3-3 is instead more often involved in the regulation of client structures and posttranslational modifications. The functional distinctness of 14-3-3 and LC8 also shows why multiple dimeric hubs are necessary.

## Conﬂict of interest

The authors declare that they have no conﬂicts of interest with the contents of this article.
